# Imaging of Wrist Injuries: A Standardized US Examination in Daily Practice

**DOI:** 10.5334/jbr-btr.1319

**Published:** 2018-01-31

**Authors:** Philippe Meyer, Pierre-Francois Lintingre, Lionel Pesquer, Nicolas Poussange, Alain Silvestre, Benjamin Dallaudiere

**Affiliations:** 1 MSK Imaging Department, Clinique du Sport de Bordeaux-Mérignac, FR

**Keywords:** Wrist, Trauma, Ultrasonography, Scaphoid, Ligaments

## Abstract

The keys to successful ultrasonography (US) of the wrist include knowledge of the relevant anatomy and understanding the biomechanical aspects. A wide spectrum of pathological findings including bone fractures (scaphoid, triquetrum) and ligament lesions (dorsal intercarpal and radiocarpal ligaments, scapholunate ligament) can be caused by an identical traumatic mechanism determined on the basis of the position of wrist at the time of injury.

In the setting of wrist trauma, an early diagnosis can minimize the potential for inappropriate or delayed treatment. We describe a practical radiological approach by using a standardized imaging protocol: standard radiographs (four views) associated with an US examination focused on seven landmarks. If there is discordance between clinical and radiological features or if the diagnosis of a disruption of the scapholunate ligament remains uncertain, additional cross-sectional imaging (MRI or CT arthrogaphy) should be performed.

## Introduction

Wrist injuries are common occurrences in sports and others traumatic conditions. The current definition of a wrist sprain is “a partial or complete ligament injury of the wrist”. However, this nonspecific term is frequently used as diagnosis in wrist injuries without taking their true underlying pathology into account. Indeed, the wrist joint is an articulation consisting of many bones and ligamentous structures which, during normal function, allows complex motions while maintaining stability.

In the coronal plane, the carp tends to dislocate towards its ulnar aspect with clenching of the fist, due to the ulnar orientation of the distal radial surface. Functionally, the Kühlmann dorsal sling is the cornerstone to avoid axial medio-carpal diastasis and is formed by the dorsal scapholunotriquetral and radiolunotriquetral ligaments [1]. In the sagittal plane, emphasis should be placed on the role of the scapholunar complex. When a compressive load is applied to the wrist, scaphoid flexion occurs due to the ascent of the trapezium [2]. In contrast, the lunatum extends during compression because of the larger size of its anterior horn. This biomechanical concept explains the predisposition to instability of the wrist. The interosseous scapholunate ligament (SLL) (especially the dorsal band which is the thickest and functionally most important part) links the bones of this complex and maintains the lunate in a state of balance between the opposing forces, allowing synchronous motion [3,4].

**Figure 1:**
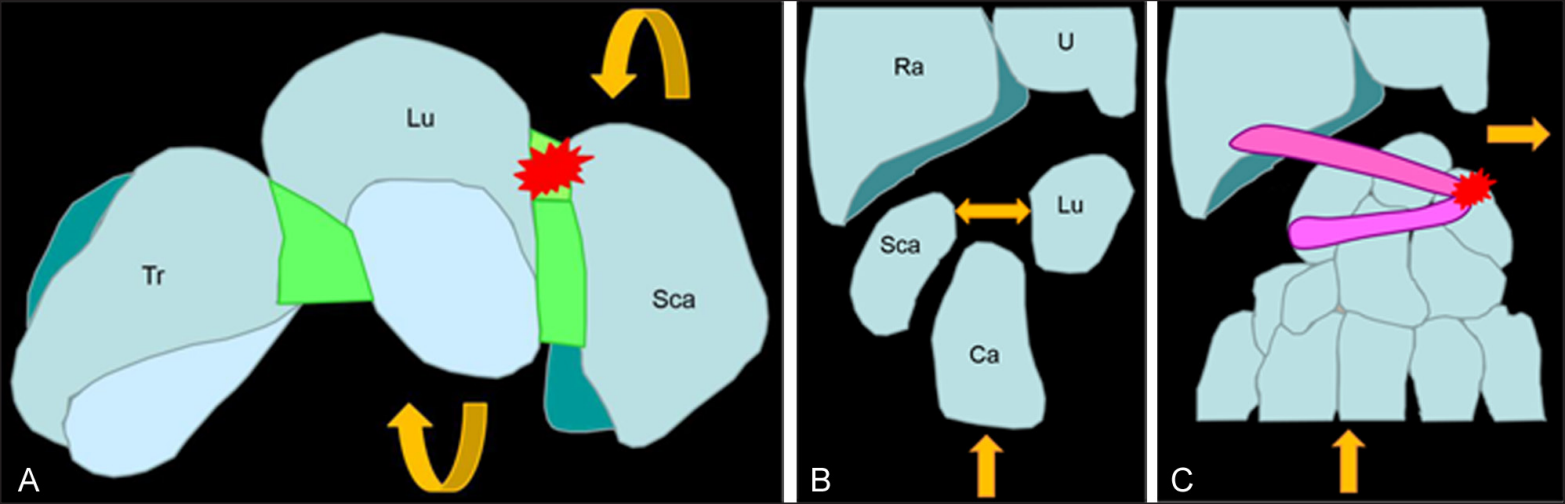
**(A–B)** The common mechanism leads to a stress of the scapholunar complex (red star) with a sudden horizontalization of the scaphoid increased by the compressive load induced by the capitates bone (rounded yellow arrow) with increased width of the scapholunate space (straight yellow arrow). **(C)** The sudden ulnar movement of the carpal bones during trauma explains the high frequency of triquetral avulsion (red star) of the dorsal sling.

The injury mechanism most commonly involves a combination of hyperextension and radial deviation stresses with an impact on the thenar eminence. This leads to a dorsiflexion of the scapholunar complex with a sudden horizontalization of the scaphoid. The commonly seen injury patterns can be caused by a similar mechanism depending on the position of the wrist on impact: a scaphoid fracture, a scapholunate disruption (exacerbated by the ascent of the capitatum), or a dorsal sling tear due to the translating movement of the carpal bones beneath the inferior margin of the radius (Figure 1A–C) [5,6,7,8].

Wrist sprains are commonly encountered but their complexity and clinical significance often go unrecognized by the medical staff or the patient himself. Awareness and detection of these injuries is crucial: scapholunate ligament tears can trigger the progression of carpal dislocation and lead to a poor long-term functional prognosis. Suboptimal or delayed patient care increases the risk for developing degenerative arthritis that may require serious therapies, especially in young and active patients [9]. Early surgical repair is associated with better functional outcomes [8,10]. In chronic injuries, the presence of arthritis usually leads to poorer outcomes. So the radiologist’s role is crucial to ensure effective early evaluation and intervention [11]. The radiological evaluation of wrist sprains requires the detection of the most clinically relevant injuries: bone fractures, disruption of the dorsal band of SLL, and tears of the dorsal sling.

The aim of this article is to propose a practical approach based on standard radiographs and US examination developed by the CARP (Cercle Aquitain de Réflexion sur le Poignet). By using this simple, reproducible, and standardized imaging protocol, radiologists can provide relevant interpretations and minimize the risk of suboptimal or delayed patient care.

## Imaging features

Scaphoid fractures represent about 76% of all carpal fractures, followed by triquetrum fractures (20%) consisting in avulsion of the common medial insertion of the dorsal sling [12]. Radiographs pertinent to better assess these fractures include: 1) posteroanterior and 2) lateral views with the wrist in a neutral position, 3) an oblique radial view 4) and then an oblique ulnar view. Others views can be realized but present a lower reproducibility (especially in patients with a limited range of motion) [13,14,15]. US examination of the wrist is an important adjunct in the initial clinical assessment and requires a high-frequency linear probe (15–17 MHz). “Hockey stick” transducers or larger linear transducers are typically used [16]. US examination has the advantages of offering the ability to examine the patient under conditions of dynamic movement and to compare the exact site of pain with the contralateral side. Herein we describe the US technique for evaluation of the ligaments and bone cortical.

**Figure 2:**
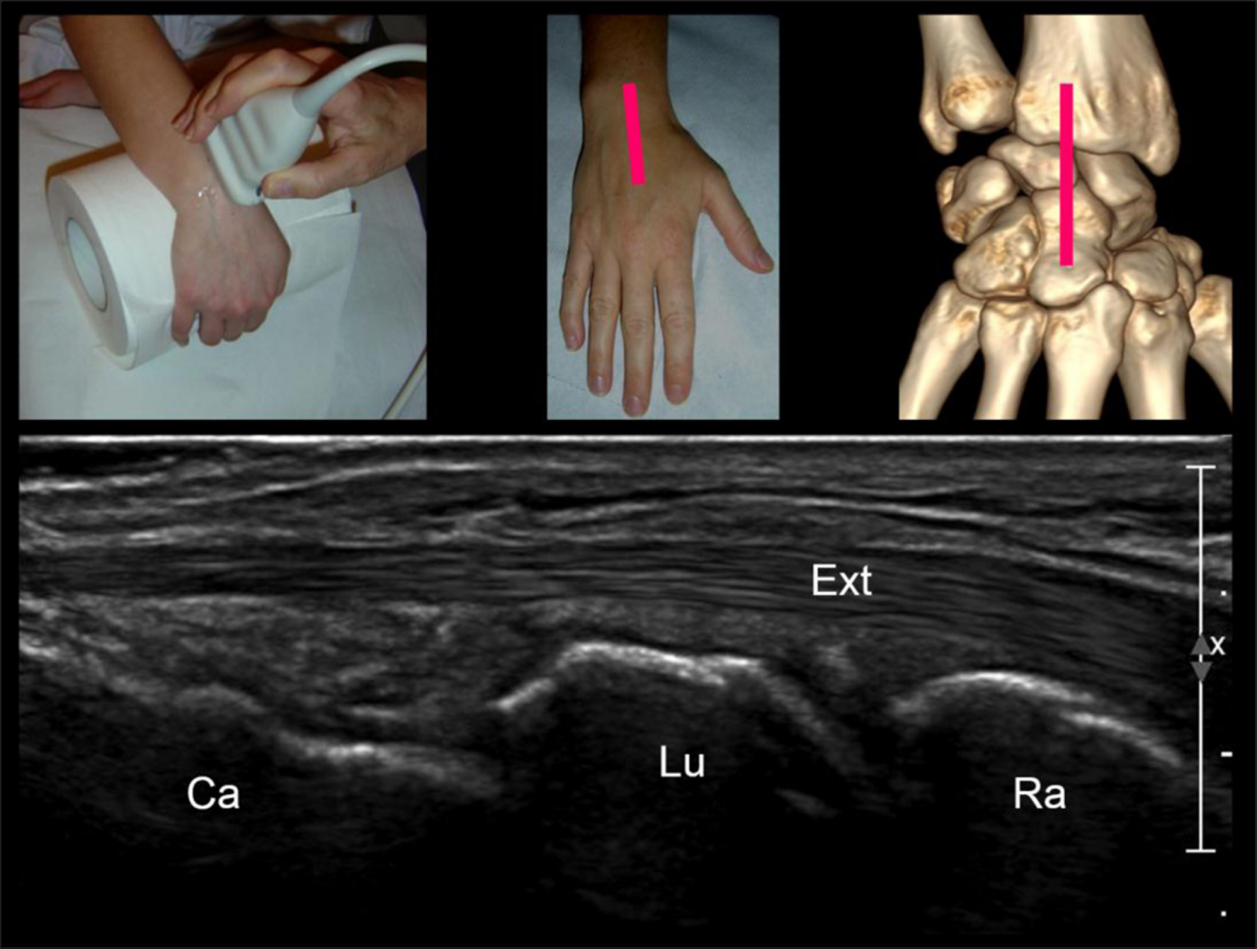
Step 1: sagittal view of the carpal bones along the long-axis of the third ray. (Ext = extensor digitorum tendon, Rad = radius, Lun = lunate, Sca = scaphoid).

**Figure 3:**
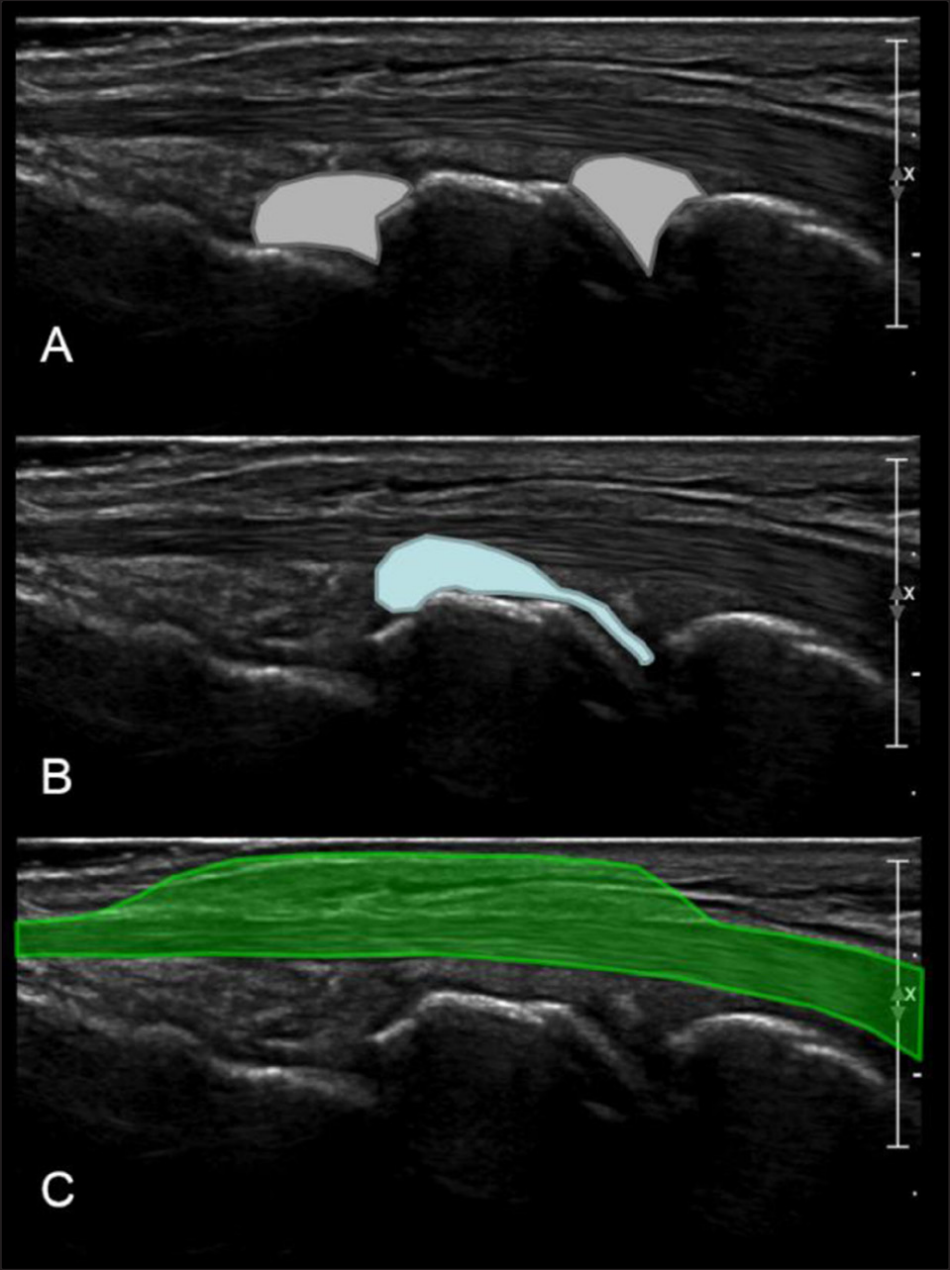
The role of the first sagittal view of carpal bones is to detect: **(A)** radiocarpal or mediocarpal joints effusions; **(B)** carpal ganglion cysts arising from the joint; **(C)** extensor tenosynovitis.

The patient is imaged while seated (his wrist resting gently on a firm pillow), with the radiologist or musculoskeletal imaging–trained sonographer seated in front of the patient. A positioning of the wrist in slight flexion or extension (by using a gel tube or a roll of paper) can be helpful. We perform a standardized wrist US examination framework that is divided into seven regional components. First step is the examination of the dorsal wrist, starting with a sagittal image of the carpal bones along the long-axis of the third ray. This view offers a general idea of the situation by showing the dorsal cortex of the distal radius, lunate and capitatum and the extensor digitorum tendons (Figure 2). This first view also allows detection of radiocarpal and mediocarpal joints effusions, an extensor tenosynovitis or a wrist ganglion arising from the scapholunate interval (Figure 3) [11,17,18].

**Figure 4:**
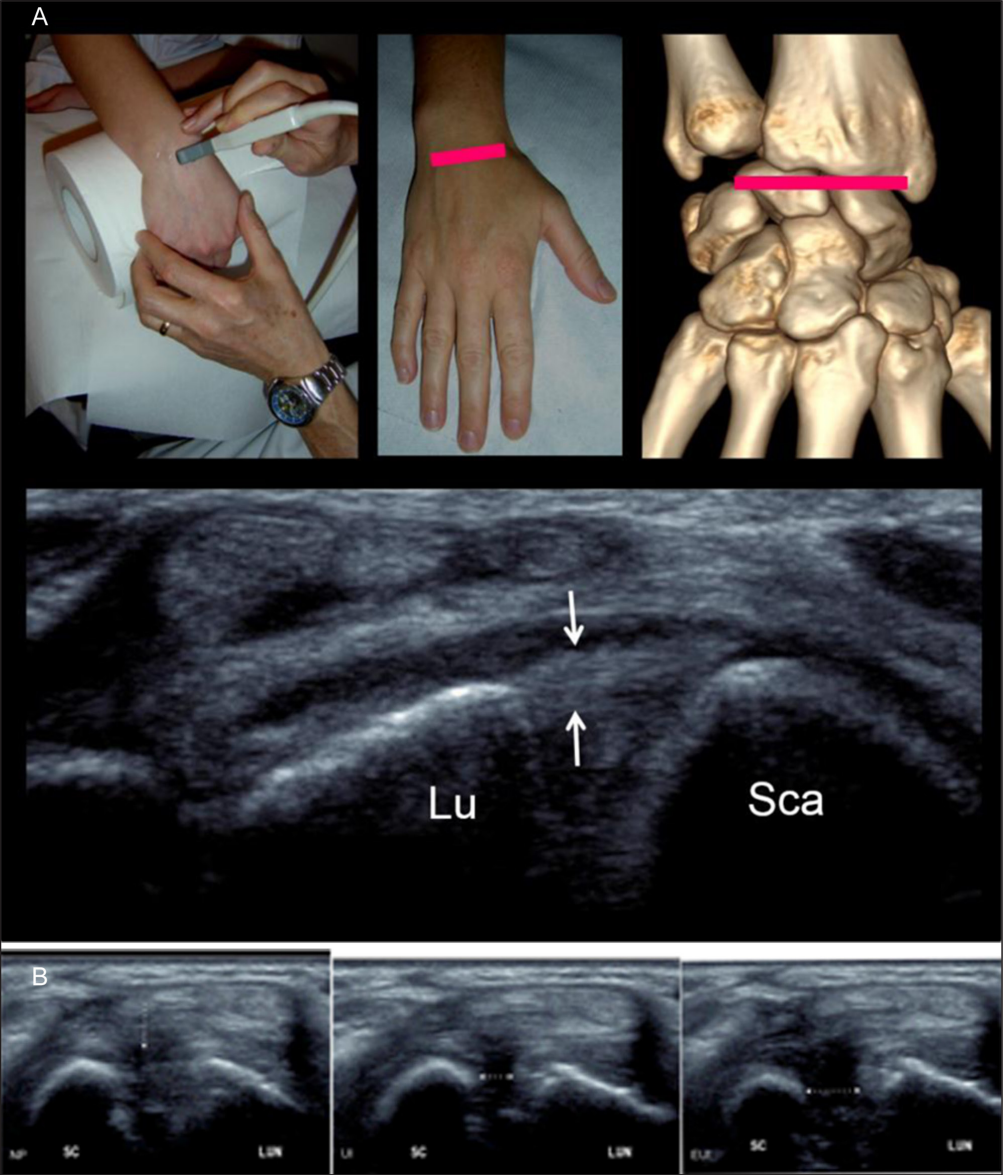
**(A)** Step 2 = axial view of the dorsal band of the scapholunate ligament. The scapholunate ligament (arrows) extends from the lunate (Lu) to the scaphoid (Sca) bones. **(B)** The aim of this second dynamic view is to look at: NP = a discontinuity of the dorsal scapholunate ligament in Neutral position (white arrow); UI = a nonvisualization of the ligament with an increased width of the scapholunate space in Ulnar Inclination (white arrow); EUI: a nonvisualization of the ligament with an increased width of the scapholunate space in Extreme Ulnar Inclination (white arrow).

The second axial view examines the dorsal band of the scapholunate ligament. After a 90° rotation, the transducer is then positioned in the transverse plane at the dorsal aspect of the distal radius, over the Lister tubercle as an anatomic landmark. This tubercle is easily and constantly palpable on the inferior radial metaphysis, between the second extensor compartment (containing the extensor carpi radialis tendons) and the third compartment (containing the extensor pollicis longus tendon). From this point, the probe is moved distally along the dorsal aspect of the proximal carpal row. The intact dorsal band of the SLL appears as a echogenic fibrilar structure mimicking a “little Achilles tendon” 1.1 mm thick (mean) and 4.2 mm long (mean) in the scapholunate interval [19]. Studies reported an almost constant visibility of the dorsal band of the SLL, but it varies depending on the equipment (tissue harmonic imaging should be used for better visualization) (Figure 4A) [20]. The aim of this second view is to search for a discontinuity or nonvisualization of the dorsal SLL or an increased width of the scapholunate space (>4 mm). Dynamic examination with ulnar and radial deviation or with clenching of the fist can be helpful for additional assessment (Figure 4B) [21].

Both the next two views explore the dorsal sling. Its proximal bundle, which is also known as the dorsal radiolunotriquetral ligament (RLTL) presents an oblique orientation originating from the distal radius at the ulnar aspect of the Lister tubercle, crossing over the lunate, and terminating on the triquetrum. For US evaluation of the dorsal RLTL, one begins by pointing the medial border of the transducer on the posterior aspect of triquetrum. The lateral border of the probe is then rotated toward the radial styloid.

**Figure 5:**
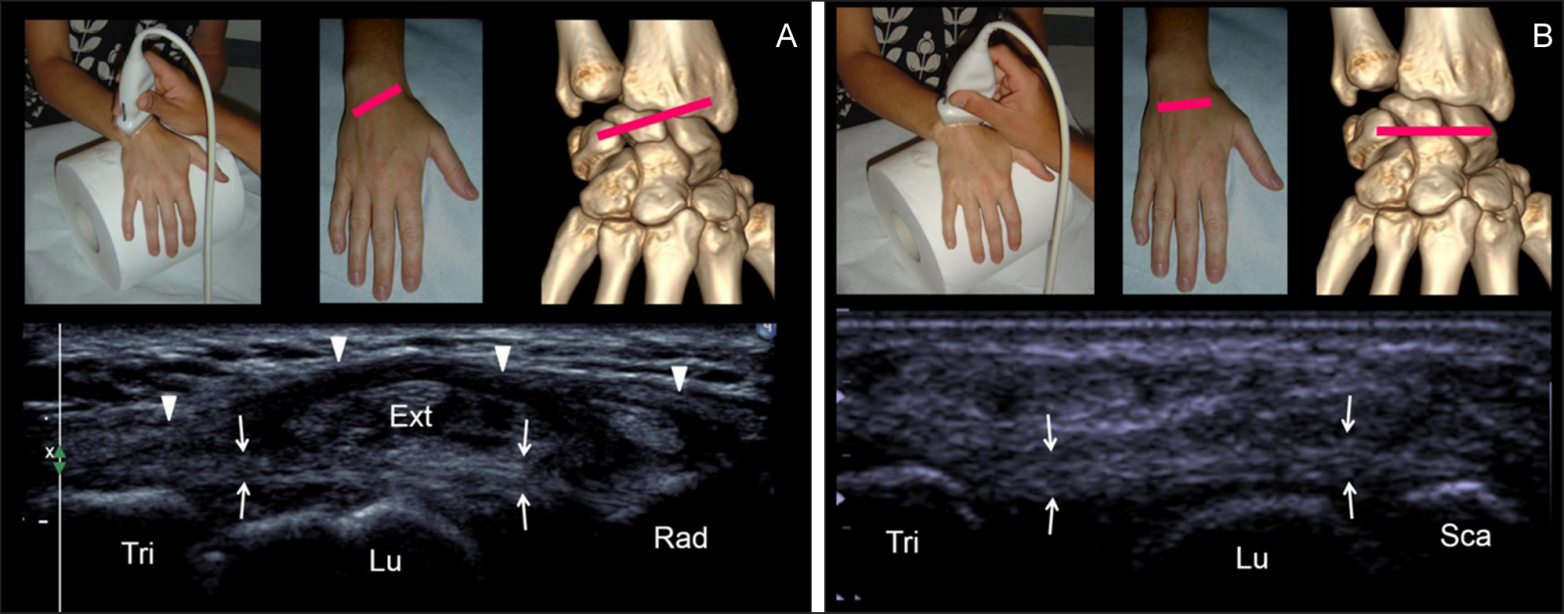
**(A)** Step 3 = Axial oblique view of the radiolunotriquetral ligament. The ligament (small arrows) extends from the radius (Rad) to the triquetrum (Tri), crossing over the lunate (Lu) without attaching. The radiolunotriquetral ligament is on continuation with the extensor retinaculum (Ext) (arrowhead). **(B)** Step 4 = Axial view of the scapholunotriquetral ligament. The ligament (arrows) extends from the scaphoid (Sca) to the triquetrum (Tri) crossing over the lunate (Lu) without attaching on.

The distal bundle, also named dorsal scapholunotriquetral ligament (SLTL), is in fact a horizontal intrinsic capsular ligament. The starting anatomic landmark is still the triquetrum. Horizontalization of the transducer (its lateral border positioning over the scaphoid) allows a good visualization of this dorsal intercarpal ligament attaching proximally on the dorsal tubercle of the triquetrum and coursing radially. US examination of the SLTL needs some experience. It should be performed with gentle toggling (i.e., rocking and angling) of the transducer to eliminate the anisotropy artifact or with the wrist positioning in slight flexion (Figure 5A, B) [5]. SLTL and RLTL cross over the lunate but do not attach on. They appear as echogenic fibrilar structures as thick as the dorsal interosseous SLL. In evaluating both bundles of the dorsal sling, emphasis should be placed on detecting a discontinuity of their fibers or an avulsion fracture of their dorsal triquetral insertions [16].

**Figure 6:**
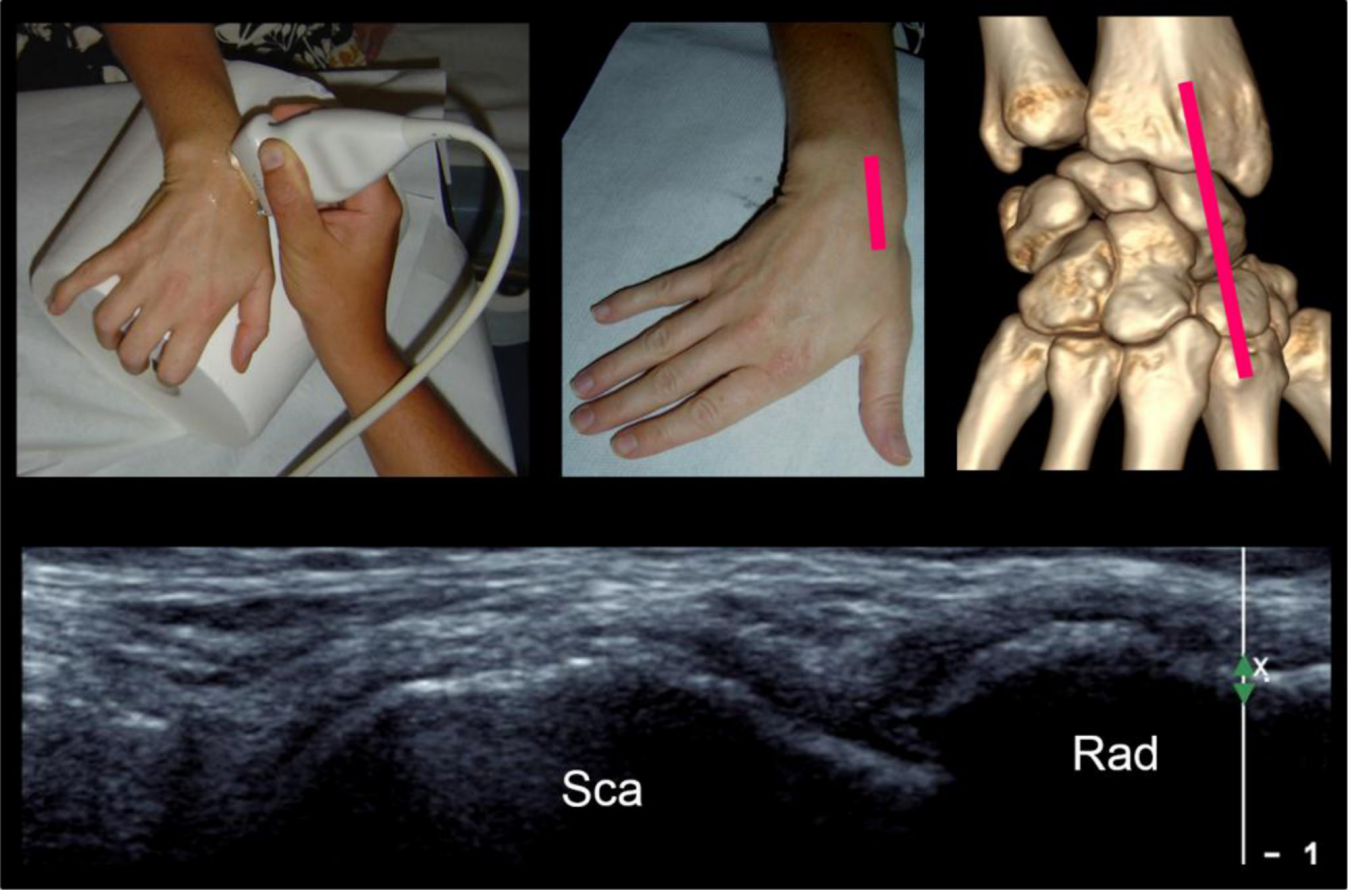
Step 5 = sagittal view of the dorsal cortex of the scaphoid with the wrist in ulnar deviation. Sca = scaphoid, Rad = radius.

The fifth step in our US examination framework is a sagittal view of the dorsal cortex of the scaphoid with the wrist in ulnar deviation in order to correctly elongate the scaphoid (Figure 6) [22,23]. The probe is placed along the long-axis of scaphoid from the radial styloid to the fifth metacarpal base. The role of this view is to detect occult scaphoid fractures (cortical disruption, subperiosteal hematoma) and radiocarpal joint effusions. US examination then focuses on the anterior and radial aspects of the wrist.

**Figure 7:**
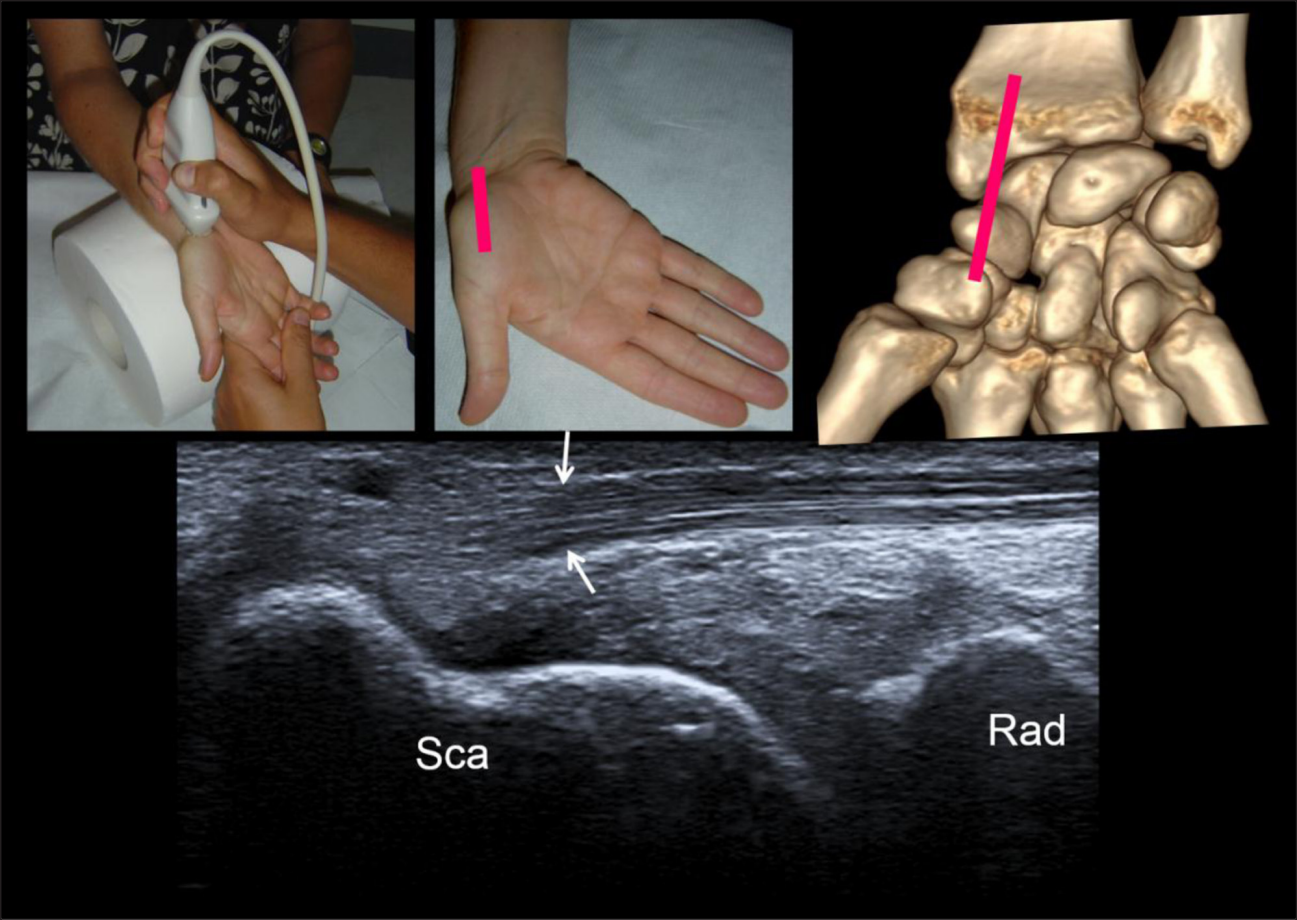
Step 6 = sagittal view of the palmar side of the scaphoid with the wrist in ulnar deviation. See the flexor carpi radialis tendon coursing anteriorly to the distal pole of scaphoid. This tendon then distally attaches on the base of the second and third metacarpals and the trapezium. Sca = scaphoid, Rad = radius.

The sixth US image is a sagittal view of the palmar border of the scaphoid with the wrist in ulnar deviation. This plane (corresponding in fact to an anterior equivalent of the fifth view), not only helps identify above-mentioned lesions (cortical disruption, subperiosteal hematoma and radiocarpal joint effusion) but also allows detection of others relevant findings including flexor carpi radialis tenosynovitis and carpal ganglion cysts arising from the anterior band of the scapholunate ligament (Figure 7) [17,24].

**Figure 8:**
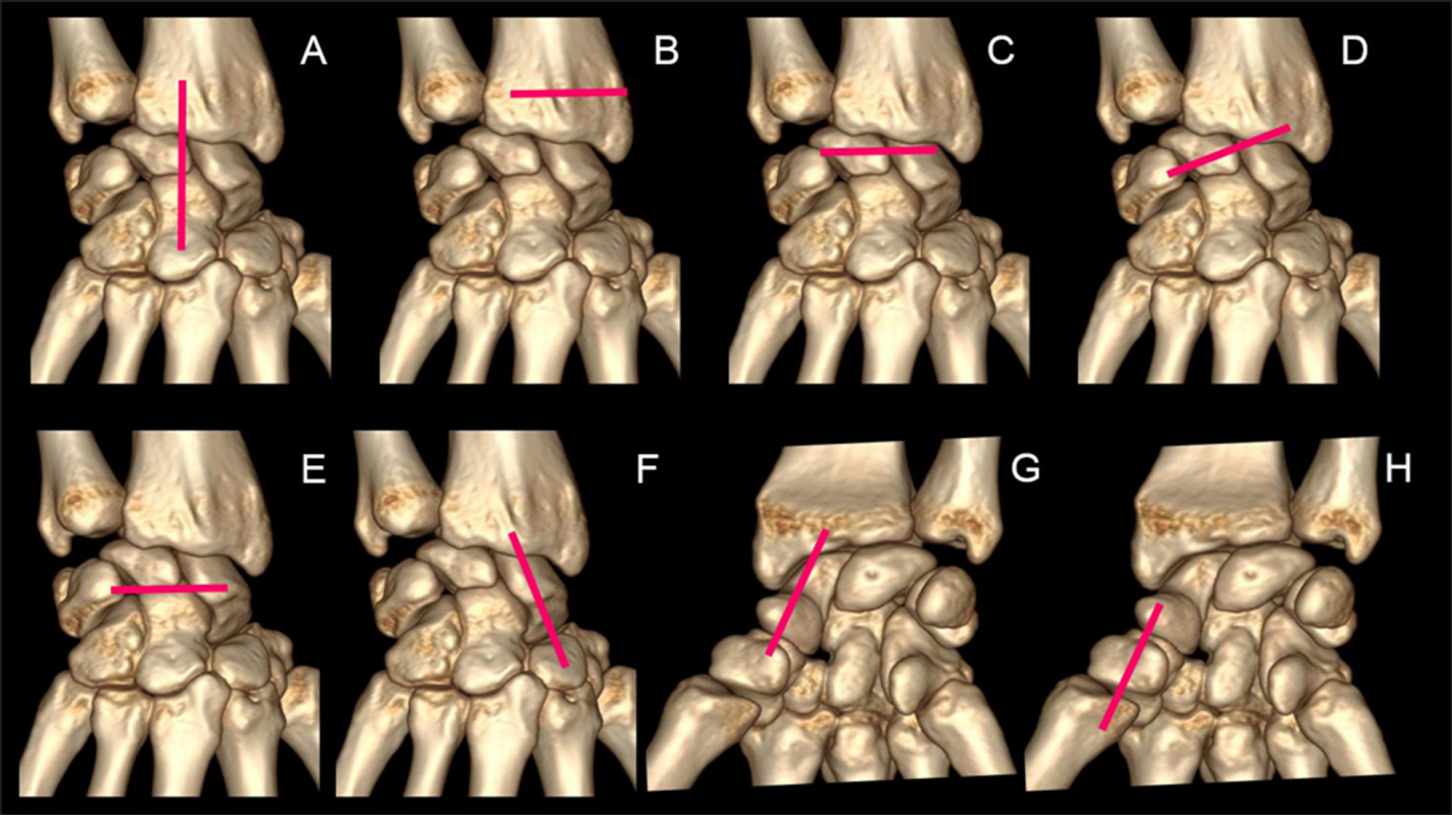
A 7-step standardized US examination framework for wrist injuries: **(A)** sagittal view of the carpal bones along the long-axis of the third ray. **(B)** localization of the Lister tubercle. **(C)** axial view of the dorsal band of the scapholunate ligament. **(D)** axial oblique view of the radiolunotriquetral ligament. **(E)** Axial view of the scapholunotriquetral ligament. **(F)** sagittal view of the dorsal cortex of the scaphoid with the wrist in ulnar deviation. **(G)** sagittal view of the palmar cortex of the scaphoid with the wrist in ulnar deviation. **(H)** sagittal view of the first ray (palmar aspect).

Standardized US examination ends with a palmar sagittal view of the first ray. From the last point, the probe is moved distally along the ventral aspect of the first metacarpal allowing exploration of the scapho-trapezoidal and the trapezo-metacarpal joints. This final view (Figure 8A–H) can also detect cortical disruptions, joint effusions, ganglion cysts, or beginning trapezo-metacarpal joint arthritis [25].

## Pathologic Conditions

**Figure 9:**
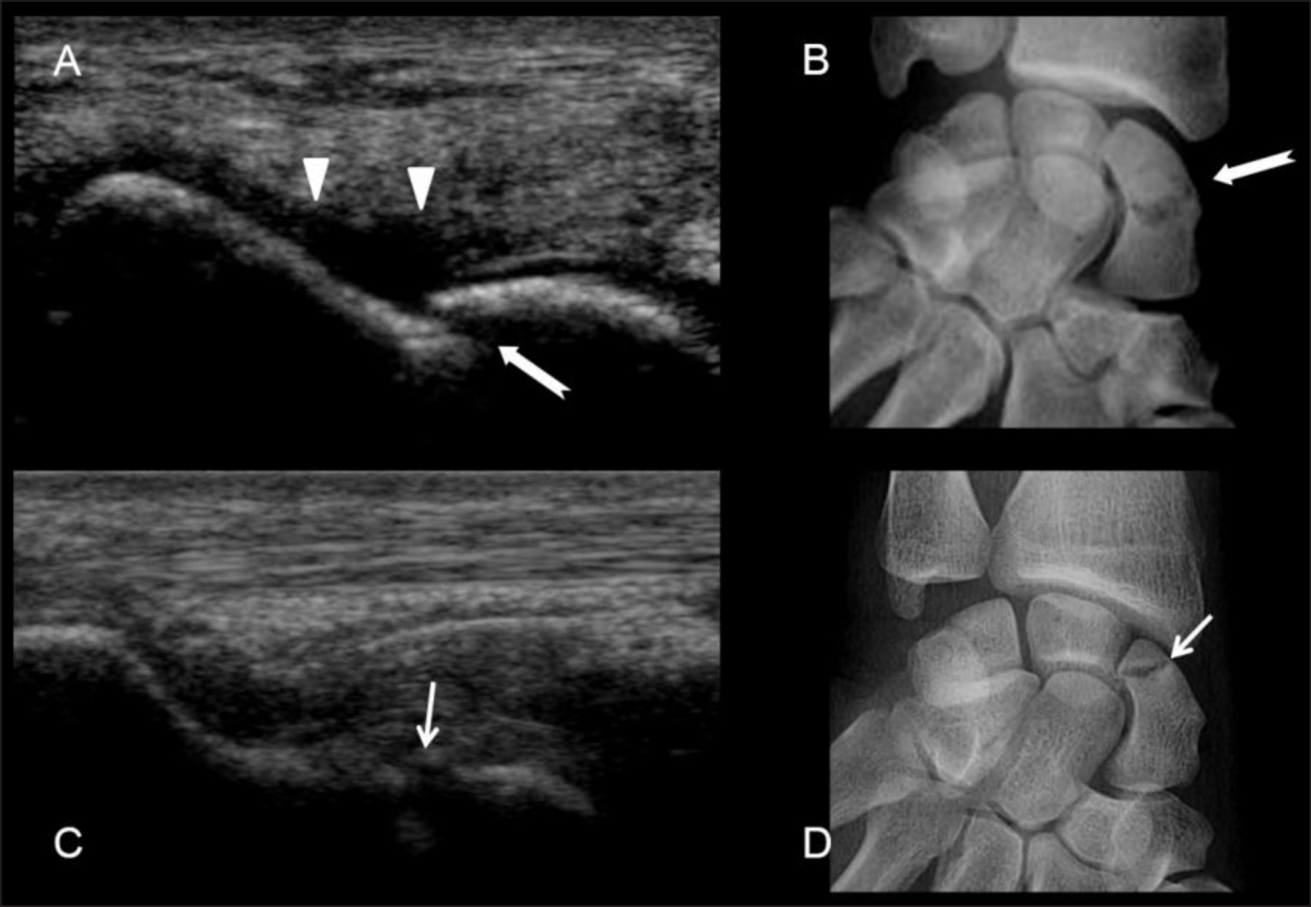
Scaphoid fractures. **(A)** and **(B)** transcortical fracture of the waist of the scaphoid. See the cortical disruption (white arrow) and the subperiosteal hematoma (arrow heads). **(C)** and **(D)** fracture of the proximal pole of the scaphoid with cortical disruption (small white arrow).

Of all the carpal bones, the most frequently fractured is the scaphoid. Schernberg classification of scaphoid fractures divides them into six types according to anatomic location of the fracture line [26]. Because the blood supply to the proximal third of the scaphoid is very tenuous (arterial flow to the scaphoid enters via the distal pole), proximal fractures are more serious and represent an increased risk of nonunion [24,26]. US examination can reliably diagnose a scaphoid fracture by showing the cortical discontinuity. However this cortical change can be discrete and a reverberation artifact may be the only US finding suggestive of a scaphoid fracture. US examination also allows detection of another, often more subtle finding that may lead to diagnosis: an elevated periosteum with subperiosteal fluid collection due to post-traumatic hematoma. Sometimes, scaphoid fractures are considered too subtle to be recognized on conventional radiographs obtained immediately after the trauma and so are only seen on US examination (also known as “occult fractures”). They may become visible during follow-up on specialized radiographic projections or on cross-sectional imaging (Figure 9) [25].

**Figure 10:**
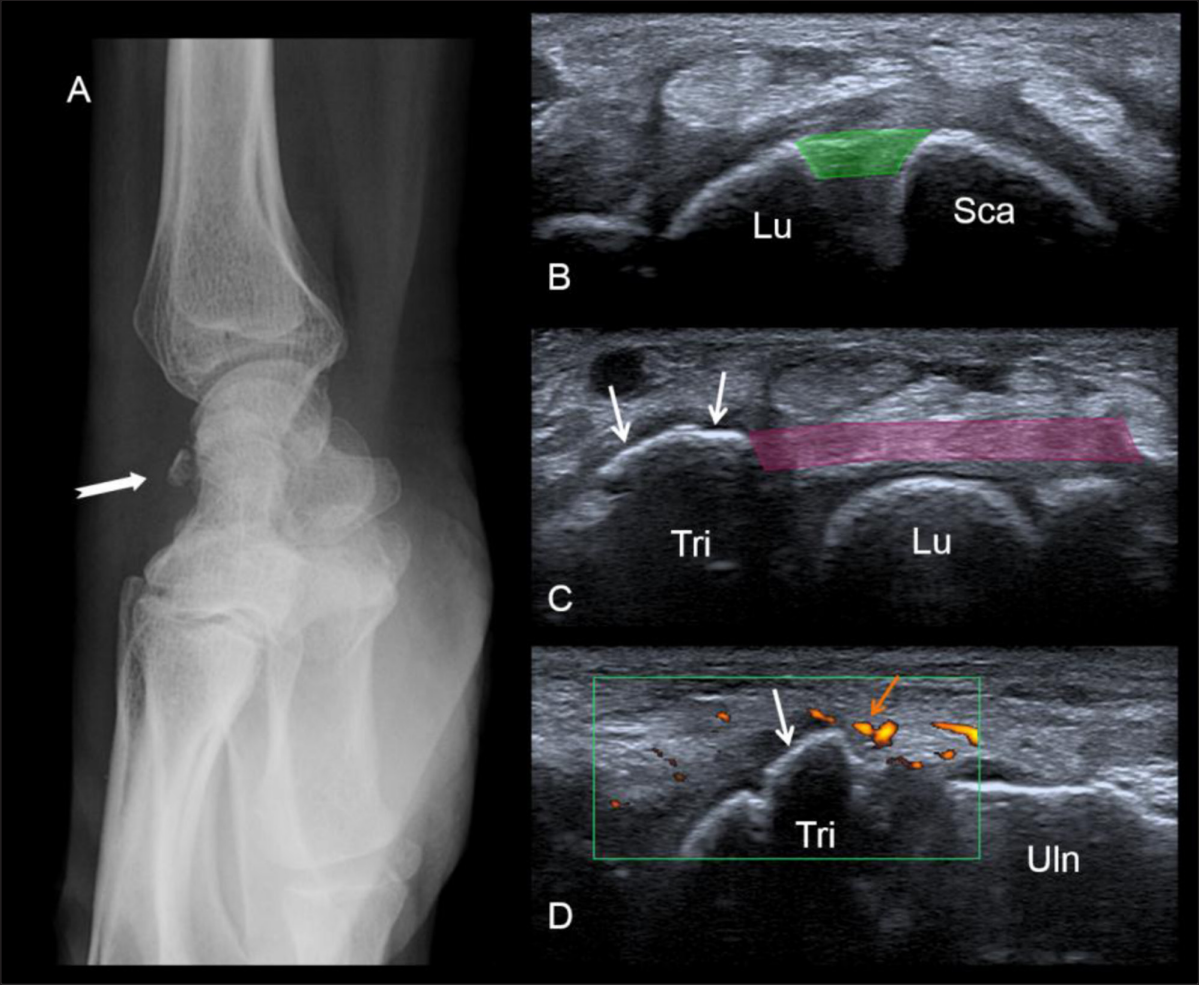
Bony avulsion of the dorsal sling (triquetral insertion). A fracture is seen on the lateral radiograph **(A)**. At the US examination, the dorsal band of the scapholunate ligament (in green) is normal **(B)**. In contrast US shows the bony avulsion of the triquetral insertion of the dorsal radiolunotriquetral ligament (white arrows). See the soft-tissue hyperemia surrounding the avulsion on the long-axis view (orange arrow). Lu = lunate, Sca = scaphoid, Tri = triquetrum, Uln = ulna.

Triquetral fractures are the second most common carpal bone fracture, after the scaphoid [5]. Such fractures result from an avulsion of the dorsal sling attachment. On plain film, dorsal avulsion injuries may be detected on a lateral projection. US examination is also a suitable tool for detection of these fractures (Figure 10).

Other carpal fractures are rare but the clinical question can be answered correctly with a focused US examination of the injured and painful anatomic structure in addition to the previous standardized views [14]. US has also the advantage of studying the ligaments and especially both the dorsal band of SLL and dorsal sling. Due to the biomechanical stress, dorsal sling injuries are almost exclusively localized in their triquetral attachments resulting either in a partial tear or in a complete disruption [8,25,26,27].

Regarding the scapholunate ligament, if a lesion is suspected, an additional cross-sectional imaging (CT- or MRI arthrography) should be systematically performed [19,20,21,28]. Indeed, in case of ligamentous lesions, the partial or complete isolated lesions of LSL are not unstable and therefore do not tend to cause loco-regional instability and osteo-arthritis. Only lesions of LSL associated with involvement of the extrinsic carp strap can cause osteoarthritis [15].

## Conclusion

Wrist fractures are treated conservatively (immobilization in plaster cast) or surgically according to their type. In the absence of fracture and if the dorsal band of the scapholunate ligament is intact, we propose a clinical follow-up: the injured wrist is immobilized by splint and a new physical examination by orthopedist is planned 10 days after the trauma. If US shows either a disruption of the dorsal band of SLL or an increased width of the scapholunate space, a surgical repair is planned. If diagnosis of SLL injury remains uncertain after the standardized evaluation, an additional cross-sectional imaging (CT- or MRI arthrography) is performed to assess the interosseous ligament.
